# Risk factors for the mental health of basic education teachers in Brazil: integrative review

**DOI:** 10.47626/1679-4435-2025-1553

**Published:** 2026-01-05

**Authors:** Ana Cristina Santiago da Costa, Evanice Avelino de Souza, Thiago Medeiros da Costa Daniele, Mirna Albuquerque Frota

**Affiliations:** 1 Universidade de Fortaleza, Programa de Pós-Graduação em Saúde Coletiva, Fortaleza, CE, Brasil; 2 Secretaria Municipal de Educação de Fortaleza, Fortaleza, CE, Brasil; 3 Centro Universitário FATENE (Unifatene), Curso de Educação Física, Caucaia, CE, Brasil

**Keywords:** mental health, teachers, Brazil, basic education, occupational stress., saúde mental, professores, Brasil, educação básica, estresse ocupacional.

## Abstract

**Introduction:**

Given the increasing incidence of mental disorders among education professionals, examining the national scientific literature on the mental health of basic education teachers in Brazil has become essential.

**Objectives:**

To assess, through an integrative literature review, the psychosocial factors associated with teachers’ mental health, with an emphasis on the prevalence of mental disorders.

**Methods:**

The search was conducted between August 2024 and January 2025 in SciELO, the CAPES Journal Portal, LILACS, and PubMed databases, using descriptors related to mental health, teachers, basic education, and Brazil, which were combined through Boolean operators AND and OR. Studies published between 2019 and 2024, available in full text in Portuguese or English, and specifically addressing the mental health of basic education teachers were included. The review was guided by the following question: what are the main risk factors that influence the mental health of basic education teachers in Brazil?

**Results:**

The analysis of 12 studies revealed a high prevalence of symptoms of anxiety, stress, and burnout syndrome among teachers. The most frequently reported psychosocial risk factors included work overload, low pay, poor working conditions, and exposure to school violence.

**Conclusions:**

Such psychosocial determinants significantly affect teachers’ mental health, characterizing a public health and occupational concern. The findings highlight the need for intersectoral policies that support teaching valuation and promote adequate working conditions and safer and healthier school environments to preserve the mental health of basic education teachers.

## Abstract (pt)

**Introdução:**

Diante do crescente número de transtornos mentais entre profissionais da educação, tornou-se necessário analisar a produção científica nacional acerca da saúde mental de professores da educação básica no Brasil.

**Objetivos:**

Avaliar, por meio de uma revisão integrativa da literatura, os fatores psicossociais associados à saúde mental desses docentes, com ênfase na prevalência de transtornos mentais.

**Métodos:**

A busca foi realizada entre agosto de 2024 e janeiro de 2025 nas bases de dados SciELO, Portal de Periódicos da CAPES, LILACS e PubMed, com base em descritores relacionados à saúde mental, professores, educação básica e Brasil, combinados utilizando os operadores booleanos AND e OR. Incluíram-se estudos publicados entre 2019 e 2024, disponíveis na íntegra, em português ou inglês, que abordassem especificamente a saúde mental de professores da educação básica. A revisão foi orientada pela seguinte questão: quais são os principais fatores de risco que influenciam a saúde mental dos professores da educação básica no Brasil?

**Resultados:**

A análise de 12 estudos evidenciou elevada prevalência de sintomas de ansiedade, estresse e síndrome de *burnout* entre os docentes. Os fatores mais associados ao adoecimento psíquico incluem sobrecarga de trabalho, baixos salários, precarização das condições laborais e violência no ambiente escolar.

**Conclusões:**

Tais fatores psicossociais impactam significativamente a saúde mental dos professores, configurando um problema de saúde pública e de trabalho. Assim, reforça-se a necessidade de políticas intersetoriais que valorizem o trabalho docente e promovam condições adequadas e ambientes escolares mais saudáveis, preservando assim a saúde mental dos professores da educação básica.

## INTRODUCTION

Teachers’ mental health has emerged as a priority issue in public health, due to the marked increase in reports of psychological distress among these professionals. International studies have shown a notably high prevalence of symptoms such as stress, anxiety, depression, and burnout syndrome in the teaching population, exceeding the rates observed in the general population.^1-4^ The World Health Organization estimates that 35% of teachers across different countries present symptoms of chronic stress, often related to adverse working conditions such as excessive workload, institutional demands, and insufficient psychosocial support.

In Brazil, the situation is equally concerning and reflects the deterioration of working conditions in basic education. Studies conducted by institutions such as Universidade de São Paulo and Universidade Estadual de Campinas indicate that approximately 60% of basic education teachers exhibit symptoms consistent with burnout syndrome. Research also shows that over 40% of these professionals have depressive symptoms, with a higher prevalence among women - who constitute the majority of the teaching workforce -, highlighting the impact of gender inequalities on teachers’ experiences of psychological distress.^5-7^

The intensification of pedagogical demands, precarious working conditions, school violence, and lack of professional valorization create a context of mental health vulnerability, contributing to increased morbidity and departure of many teachers from the profession.^8,9^ Recent data suggest that about 40% of Brazilian teachers present signs of emotional exhaustion, and leaving the career has been frequently adopted as a strategy of self-preservation.^10,11^ This phenomenon exacerbates the shortage of qualified professionals in the educational system and undermines the continuity and quality of teaching and learning processes.

Given this scenario, teachers’ mental health should be understood as a key determinant of educational quality and social justice. Their psychological well-being directly influences their ability to foster healthy school environments, engage students, and cope with the daily demands of teaching. The production of knowledge on this topic is thus crucial to support public policies and institutional strategies that promote mental health and strengthen the professional value of education professionals.

In this context, the present study aimed to analyze the scientific evidence on the mental health of basic education teachers in Brazil, focusing on the prevalence of and factors associated with mental disorders.

## METHODS

This integrative literature review examines the mental health of basic education teachers in Brazil, with the purpose of synthesizing evidence to support decision-making and improve practices in the field of public health, as well as to identify gaps for future research.^12,13^

The six methodological steps recommended for integrative review were followed^13^: (i) formulation of the research question; (ii) definition of inclusion and exclusion criteria; (iii) selection of databases and development of the search strategy; (iv) selection of studies; (v) data extraction and analysis; and (vi) critical appraisal of the included studies, discussion of the findings, and synthesis of the evidence.

The research question was formulated using the PICO framework (P: population; I: intervention of interest; C: context; O: outcome), adapted for qualitative/contextual reviews. In this study, the PICO components were defined as follows: P - basic education teachers in Brazil; I - psychosocial and occupational risk factors; C - the school environment. Based on this, the following review question was formulated: what are the main risk factors that influence the mental health of basic education teachers in Brazil?

Descriptors and free-text terms were used, combined with Boolean operators: “*saúde mental*” AND (“*professor*” OR “*docente*”) AND (“*Educação Básica*” OR “*Ensino Fundamental*” OR “*Educação Infantil*” OR “*Ensino Médio*”) “*Brasil*,” as well as their English equivalents, with no time restrictions.

Data were collected from August 2024 to January 2025 and later reviewed and updated in June 2025. The Boolean operators AND and OR were applied to combine the descriptors first in pair and then in triads, with no time restrictions.

Searches were conducted in the following databases: Latin American and the Caribbean Health Sciences Literature (Literatura Latino-Americana em Ciências da Saúde, LILACS), Scientific Electronic Library Online (SciELO), CAPES Periodicals Portal, and PubMed. These databases were selected because they represent important sources of knowledge with worldwide coverage of scientific journals, according to the syntax presented in Table 1.

**Table 1:** Summary of the descriptors and Boolean operators used in the database search, Fortaleza, CE, Brazil, 2025

Databases	Syntax used (n)
Latin American and the Caribbean Health Sciences Literature (Literatura Latino-Americana em Ciências da Saúde, LILACS)	Saúde Mental, [AND], Professor, [OR], Docente (n = 591) Saúde Mental, [AND], Ensino (n = 100) Saúde Mental, [AND], Educação Básica (n = 39) Saúde Mental, [AND], Escola (n = 51)
Scientific Electronic Library Online (SciELO)	Saúde Mental, [AND], Escola (n = 504) Saúde Mental, [AND], Ensino (n = 393) Saúde Mental, [AND], Docente (n = 47) Saúde Mental, [AND], Professor (n = 30)
CAPES Periodicals Portal	Saúde Mental, [AND], Professor (n = 302) Saúde Mental, [AND], Professor, [OR], Escola (n = 232) Saúde Mental, [AND], Professor, [OR], Docente (n = 230) Saúde Mental, [AND], Professor, [OR], Ensino Fundamental (n = 102) Saúde Mental, [AND], Professor, [OR], Ensino Médico (n = 90) Saúde Mental, [AND], Professor, [OR], Educação Básica (n = 88) Saúde Mental, [AND], Professor, [OR], Educação Infantil (n = 33)
PubMed	Mental Health, [AND], School, [AND] Brazil (n = 78) Mental Health, [AND], Teachers, [AND] Brazil (n = 37)

**Figure 1:**
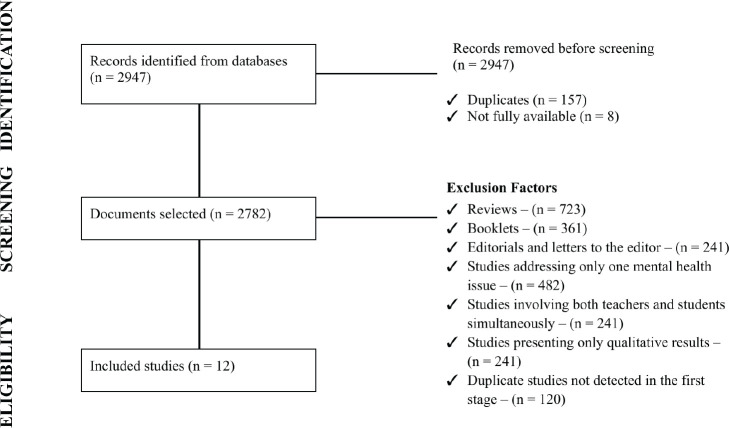
Flowchart of the search, exclusion, and selection process of retrieved articles.

The study selection process followed the guidelines of the Preferred Reporting Items for Systematic Reviews and Meta-Analyses (PRISMA) and is illustrated in the flowchart shown in Figure 1. Data extraction used a standardized instrument containing the following information: author, year, objective, study type and design, population evaluated, data collection instruments, main findings, and risk factors associated with mental health. Screening and extraction were performed independently by two reviewers, and any disagreements were solved by consensus.

Eligible studies comprised primary studies available in full-text, published from January 2019 to June 2024, in Portuguese or English, and specifically addressing the mental health of basic education teachers. Excluded materials included systematic, narrative, or integrative reviews, booklets, editorials, letters to the editor, and duplicate studies.

## RESULTS

The present review identified 30 articles, and its exclusion criteria and all steps conducted up to the final stage are described in Figure 1.

Among the 12 studies included, 1 was conducted simultaneously in different regions of Brazil, whereas 2 were conducted in the Northeast region (Rio Grande do Norte and Alagoas), 4 in the Southeast region (Minas Gerais and São Paulo), and 5 in the South region (Rio Grande do Sul and Santa Catarina). Additional characteristics of the studies, such as publication year, location, and sample, are described in Table 2.

**Table 2:** Studies investigating the prevalence of factors associated with the mental health of basic education teachers (n = 12)

Authors	Year	City (state)	Population	Sample
Magalhães et al.^14^	2023	Montes Claros (MG)	634 BE public school teachers	Men - NI; women - 85.3% Mean age: 40.6 years
Silva et al.^15^	2023	Five municipalities in the state of Rio Grande do Sul	249 BE public school teachers	Men - 40 (16.1%); women - 161 (64.7%); did not answer - 48 (19.2%) Age - from 36 to 40 years
Herbstrith et al.^16^	2023	Bagé (RS)	27 BE public school physical education teachers	Men - 14 (51.9%); women - 13 (48.2%) Mean age - 47.7 years (from 28 to 65 years)
Silva et al.^17^	2023	NI (RN)	273 BE public school teachers	Men - 84 (30.8%); women - 189 (69.2%) Age - from 21 and 76 years
Kobarg^18^	2023	Itajaí (SC)	40 BE public school teachers	Men - 14 (34.2%); women - 27 (65.8%) Agee - over 30 years.
Ribeiro et al.^19^	2023	Different regions of Brazil	499 BE public and private school teachers	Men - 108 (21.6%); women - 391 (78.4% ) Mean age - 39.4 years
Rodrigues et al.^20^	2022	Montes Claros (MG)	82 BE public school teachers	Men - 8 (9.8%); women - 74 (90.2%) Mean age - 43 years (from 23 to 64 years)
Alves et al.^21^	2022	Arapiraca (AL)	219 BE public school teachers	Men - 38 (17.4%); women - 181 (82.6%) Mean age - 41.9±28.3 years
Deffaveri et al.^22^	2020	Municipality in northern Rio Grande do Sul	200 BE public and private school teachers	Men - 35 (17.5%); women - 165 (82.5%) Mean age - 40.75 years
Gasparin & Wagner^23^	2020	Municipality in northern Rio Grande do Sul	109 BE public and private school teachers	Men - 4 (3.7%); women - 105 (96.3%) Age - 42.06±9.38 (from 23 to 62)
Machado & Limongi^24^	2019	Uberlândia (MG)	330 BE public school teachers	Men - NI; women - (88.2%). Age - 43.5±9.73 years (from 22 to 70 years)
Costa & Silva^25^	2019	Municipality in the region of Vale do Paraíba (SP)	105 BE public school teachers	Women - 105 Age - from 21 to 60 years

AL = Alagoas; BE - basic education; MG = Minas Gerais; NI = not informed. RN = Rio Grande do Norte; RS = Rio Grande do Sul; SC = Santa Catarina; SP = São Paulo.

**Figure 2:**
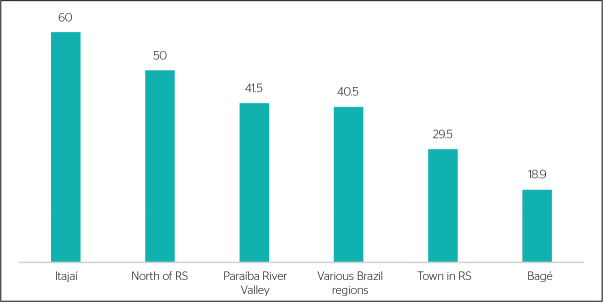
Prevalence (%) of anxiety symptoms among basic education teachers in Brazil.

Regarding the methods used, most studies employed questionnaires,^14,15,17,18,21,22,24^ with no predominance of any instrument. With respect to mental disorders, a high prevalence of anxiety^16,18,19,22,23,25^ was observed in the studies included in this review, as illustrated in Figure 2.

RS = State of Rio Grande do Sul

Concerning the factors associated with mental health, low pay,^19,25^ violence from students or their parents,^19,24,25^ and excessive workload^14,19,16,24^ were the most prominent aspects identified in the studies analyzed. Table 3 describes the instruments applied and the main results of each study included in the present review.

**Table 3:** Prevalence of mental disorders and factors associated with mental health among basic education teachers in Brazil (n = 12)

Author	Instruments	Mental disorders	Associated factors
Magalhães et al.^14^	CESQT and BDI	14.5%, 24.0% and 19.3% of teachers experienced symptoms such as burnout syndrome, depression, and voice disorders, respectively.	Female sex Longer working week Negative self-perception of health
Silva et al.^15^	Questionnaire adapted from the Mental Health and Work Protocol	47.8% perceived themselves to be emotionally exhausted, and 71.5% pointed to stress in the work environment.	Lack of appreciation by society
Herbstrith et al.^16^	HADS	25.9% and 18.9% of teachers possibly had depression and anxiety, respectively.	High levels of emotional exhaustion Heavy class load
Silva et al.^17^	SRQ-20	56.8% of teachers had a CMD.	Employment with the State
Kobarg^18^	MHI-5	61% of the sample were at a moderate level of depression and anxiety.	Being married Having children Working in the classroom
Ribeiro et al.^19^	HADS	40.5% and 23.4% of teachers had probable anxiety and depression scores, respectively.	Poor work environment Working for more than 40 h Inadequate physical space Inadequate income Violence caused by students
Rodrigues et al.^20^	PHQ-9	48.8% of teachers presented depressive symptoms.	Vocal disorders and lack of vocal warm-up
Alves et al.^21^	CES-D and WHOQOF-bref	35.6% of teachers were at risk of depression.	The environment domain of quality of life
Deffaveri et al.^22^	Sociodemographic and health questionnaire, LIS-A, LIS-E, and DASS-21	50% of public school teachers had stress symptoms.	Lack of physical activity Hypertension
Gasparin & Wagner^23^	DASS-21	21.8%, 29.5% and 28.2% of public school teachers had depression, anxiety, and stress scores ranging from moderate to extremely severe, respectively.	Socio-educational skills were related to depression symptoms in private school teachers. Heavy class load and limited material and resources in public schools
Machado & Limongi^24^	GHQ-12	43.9% of the sample presented a CMD.	Experience with school violence Working double shifts Female sex Use of sleep disorder drugs Permanent employment relationship
Costa & Silva ^25^	BAI and BDI	41.5% of teachers exhibited mild, moderate or severe levels of anxiety, and 30.5% exhibited mild or moderate levels of depression.	Dissatisfaction with structural factors (salary, rooms, and materials) and with relation factors (students’ and their parents’ behavior).

BAI = Back Anxiety Inventory; BDI = Beck Depression Inventory; CES-D = Center for Epidemiologic Studies Depression Scale; CESQT = Questionnaire for the Assessment of Burnout Syndrome (Cuestionario para la Evaluación del Síndrome de Quemarse por el Trabajo); CMD = common mental disorder; DASS-21 = Depression, Anxiety and Stress Scale; GHQ-12 = General Health Questionnaire-12; HADS = Hospital Anxiety and Depression Scale; LIS-A = Anxiety Symptom Intensity Survey (Levantamento de Intensidade de Sintomas de Ansiedade); LIS-E = Stress Symptom Intensity Survey (Levantamento da Intensidade de Sintomas de Estresse); MHI-5 = Mental Health Inventory-5; PHQ-9 = Patient Health Questionnaire-9; SRQ-20 = Self-Reporting Questionnaire-20; WHOQOL-bref = World Health Organization Quality of Life Brief.

## DISCUSSION

The findings of this integrative review revealed a high prevalence of anxiety symptoms among basic education teachers, corroborating results from previous studies conducted in the Brazilian context.^15,26-30^ Despite the increased visibility of this topic in the national literature in recent years, there is still a shortage of studies that systematically and thoroughly explore the factors associated with the psychological distress affecting these professionals.

Several contextual elements contribute to this scenario, particularly the geographic location of schools. Many public schools are located in peripheral and remote areas that frequently exhibit high levels of urban violence and the presence of criminal factions.^31,32^ Under such conditions, teachers confront not only pedagogical demands but also daily situations of insecurity, resulting in a hostile and emotionally exhausting work environment. Continuous exposure to risks and perception of vulnerability negatively affect physical and emotional well-being, contributing to the worsening of stress and anxiety symptoms.

In addition to safety concerns, the precariousness of school infrastructure constitutes an important determinant of psychological distress. The absence of basic facilities, such as rest areas, appropriate restrooms, locker rooms, and spaces designated for meals or breaks, undermines quality of life and hinders physical and emotional recovery throughout the workday.^33,34^ In many situations, teachers spend extended periods at school without access to conditions that facilitate the relief of accumulated tension, which contributes to progressive exhaustion.

Although these aspects were not directly analyzed by all the studies included, the recurrence of these findings suggest an interrelationship between structural precariousness, everyday insecurity, and teachers’ mental distress. This body of evidence reinforces the need for public policies aimed not only at professional valorization, but especially at the implementation of structural improvements to promote safe, supportive, and healthy school environments.

In addition to location and infrastructure, other factors were associated with teachers’ mental health, such as low pay, violence from students and their families, and excessive workload. Low pay has been identified as a vulnerability factor, as it compels many teachers to work at several institutions, reducing the time available for rest and leisure and intensifying the feeling of professional devaluation.^27^

School-based violence, whether verbal or physical, constitutes another mental health risk factor, since teachers who experienced aggression showed higher prevalence of anxiety and depression symptoms.^35^ This type of violence affects physical integrity and, above all, psychological and emotional safety, generating fear, insecurity, and reduced professional motivation.

Excessive workload, often associated with long working hours, multiple administrative and pedagogical responsibilities, and pressure to meet performance goals, is among the factors most widely identified as harmful to teachers’ mental health. This scenario highlights the need for institutional strategies that prioritize balanced management of demands and the promotion of well-being.^36^

In light of these findings, this review underscores the importance of public policies focused on promoting teachers’ mental health, which encompasses improvements in working conditions, professional recognition, and access to psychological support in educational institutions. The analysis showed that the majority of studies reviewed were conducted predominantly in public schools, possible due to increased exposure of professionals to adverse working conditions, such as excessive workload, low pay, and inadequate infrastructure.

A predominance of women in the samples was also identified, reflecting the substantial female participation in teaching, particularly in the early stages of basic education, which points to the need of incorporating gender perspectives into analyses of psychological distress. Another relevant aspect was the variety of instruments to assess mental health which, although contributing to a multidimensional understanding of the phenomenon, hinders direct comparisons across studies and the generalization of findings. This reinforces the need for methodological standardization in future research.

This study presented several limitations, including methodological heterogeneity across the analyzed papers, particularly with respect to the mental health assessment instruments and the variety of research designs employed. Such variability hinders direct comparisons of results and limits the feasibility of quantitative synthesis. Furthermore, some studies relied on small samples or samples restricted to specific geographic contexts, which may constrain the generalizability of the results to other school settings. It is also noteworthy that most investigations employed cross-sectional designs, making it impossible to establish causal links between the identified risk factors and the occurrence of symptoms of anxiety or other mental disorders.

Finally, potential publication bias should be considered, since studies with statistically significant findings are more likely to published, which may influence the overall understanding of the phenomenon. These limitations highlight the need for further research with representative samples, standardized methodologies, and longitudinal approaches that can provide deeper insight into the mental health of basic education teachers.

## CONCLUSIONS

The findings of this integrative review reveal a concerning scenario regarding the mental health of basic education teachers in Brazil. The high prevalence of symptoms such as anxiety, stress, and burnout reflects the workload burden experienced by these professionals in their daily routine, characterized by precarious working conditions, professional devaluation, and school violence. These factors affect not only teachers’ individual health but also the functioning of educational institutions and, consequently, the teaching-learning process.

In light of this scenario, the development of public policies that address the structural causes of teacher illness is imperative. There is a need to ensure decent working conditions, equitable pay, institutional support, and mental health care strategies directed at the teaching professional. Furthermore, the strengthening of preventive measures against psychological distress and the promotion of occupational health programs specifically designed for the school environment.

Finally, it is important to highlight the need for further research that expands the understanding of the social and institutional determinants of teachers’ mental health, particularly through qualitative and community-based approaches. Addressing this issue requires not only the production of scientific knowledge but also the engagement of managers, health care professionals, and civil society to construct a more welcoming, safe, and health-promoting school environment.
